# Effects of diluents on cell culture viability measured by automated cell counter

**DOI:** 10.1371/journal.pone.0173375

**Published:** 2017-03-06

**Authors:** Aaron Chen, Matthew Leith, Roger Tu, Gurpreet Tahim, Anish Sudra, Swapnil Bhargava

**Affiliations:** 1 BioProcess Development, Seattle Genetics, Inc., Bothell, Washington, United States of America; 2 Clinical Manufacturing, Seattle Genetics, Inc., Bothell, Washington, United States of America; The Ohio State University, UNITED STATES

## Abstract

Commercially available automated cell counters based on trypan blue dye-exclusion are widely used in industrial cell culture process development and manufacturing to increase throughput and eliminate inherent variability in subjective interpretation associated with manual hemocytometers. When using these cell counters, sample dilution is often necessary to stay within the assay measurement range; however, the effect of time and diluents on cell culture is not well understood. This report presents the adverse effect of phosphate buffered saline as a diluent on cell viability when used in combination with an automated cell counter. The reduced cell viability was attributed to shear stress introduced by the automated cell counter. Furthermore, length of time samples were incubated in phosphate buffered saline also contributed to the observed drop in cell viability. Finally, as erroneous viability measurements can severely impact process decisions and product quality, this report identifies several alternative diluents that can maintain cell culture viability over time in order to ensure accurate representation of cell culture conditions.

## Introduction

Rapid, accurate and precise assessment of cell culture viability (viability) is critical to industrial cell culture process development and manufacturing of the monoclonal antibody (mAb)-based therapeutic proteins. Viability provides not only information on process performance and reproducibility, but also a basis to calculate other important parameters such as viable cell density (VCD) [1]. Maintaining a desirable viability profile throughout the cell culture process has become progressively important to enhance protein production. The observed increases in mAb production in recent years is partly attributed to the increased understanding of cell engineering and process optimization to achieve and sustain high VCD throughout the culture duration [2,3]. The importance of cellular viability is beyond protein yield as it is also a critical parameter for maintaining protein quality. Intracellular enzymes, which could be released upon cell death, were found to be responsible for either modifying the glycans [4] or reducing the inter-chain disulfide bonds of immunoglobulin G subclass 1 (IgG1) [5,6]. Therefore, viability is often incorporated as part of the harvest criteria, due to the potential impact upon protein quality.

Trypan blue dye-exclusion microscopy, a permeability assay, is the gold standard for measuring cell viability and density due to its simplicity and accessibility [7,8]. The permeability assays are based on cell membrane integrity. The compromised cell membranes allow vital stains, which are normally excluded by functional cell membranes, to freely traverse into cytoplasm and stain the dead cells. Dead cells appear blue, and the live cells appear translucent under bright-field microscopy. However, this highly subjective method is prone to human error if the analysts are not well-trained, and the counting procedure is time consuming.

To address these issues, commercially available automated cell counters utilizing trypan blue dye-exclusion microscopy and digital image processing have been developed [9]. The automated cell counter mixes cell culture samples with the dye, and passes the mixture through a flow cell where digital images are captured. Human error is reduced due to the automated sample handling and minimized user manipulation. Throughput is increased due to image processing software which enables rapid quantitation and differentiation of live and dead cells within minutes. In addition, multiple samples can be loaded and processed sequentially.

However, automated cell counters require sample dilution when the total cell counts (TCC) of the sample exceed a certain value in order to maintain counting precision and accuracy. Furthermore, sample dilution has become a common practice to preserve vessel working volume in microscale bioreactor (MBR) systems.

Recent advancement in high throughput microscale bioreactor (MBR) systems allow bioprocess developers to effectively pursue large design of experiment studies while maintaining data quality comparable to those generated from bench-top stirred-tank bioreactor (STBR) systems [10–14]. These MBRs have become increasingly important to accelerate cell culture process development as well as support of quality by design initiatives. The working volume for such systems typically ranges from hundreds of micro- to tens of milli-liter scales. To preserve the working volume, selective sampling in conjunction with sample dilution is a common practice.

This report presents the adverse effect of, and alternatives for, cell culture samples diluted in phosphate buffered saline (PBS). Lower viability and greater variability was observed with PBS diluted samples. Furthermore, the viability of PBS diluted samples continuously decreased over time and at a faster rate than the other conditions. This phenomenon was observed with multiple cell lines and different culture systems. The decrease in viability was attributed to shear stress introduced during sample preparation by the automated counter, and can be mitigated by using shear-protectant containing diluents. Inaccurate viability measurements can significantly impact process decisions such as feeding strategy and harvest timing. Therefore, care needs to be taken when preparing viability samples with diluents to ensure the results are accurate and representative of the culture.

## Materials and methods

### Cell culture

**Fig 1:**
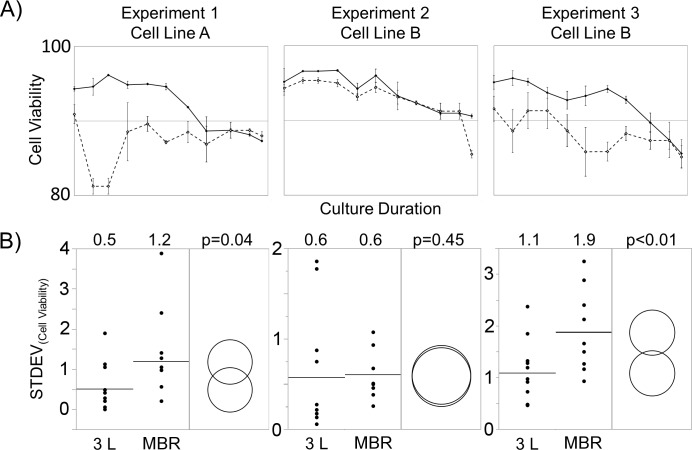
(A) Viability traces over time between the MBR (dashed lines) and 3 L bioreactor (solid lines) systems. Traces represent the means of replicates (n = 3 per experiment), and error bars represent standard deviations. (B) Daily standard deviations (STDEV, n = 11). Bars and the numbers above graphs represent the means of daily standard deviations, and comparison circles represent the results using Tukey-Kramer honest significant difference (Tukey) test. Raw data included in S1 Dataset.

Industrial-relevant Chinese hamster ovary (CHO) cell lines were used in this study. The cell lines were genetically engineered to secrete recombinant mAbs. Cells were cultured and maintained in commercially-available proprietary chemically-defined basal medium. Unless otherwise specified, cell line B (Fig 1A) was used to generate most of the data described in this report. Cells were cultured in 250 mL non-baffled Erlenmeyer flasks (Corning Inc., Corning, NY) using batch process. The cell culture process conditions for the shake platform were 37°C, 125 rpm (with 19 mm throw) in a humidified incubator with 5% CO_2_.

### Sample dilution

Cell culture samples were either not diluted (neat) or diluted with various diluents such as fresh medium, PBS (P/N: 10010–031, Thermo Fisher Scientific, Grand Island, NY), PBS with biological buffers (PBS+Buffer) and PBS with shear protectant (PBS+Shear Protectant). To create PBS+Buffer, 2.2 g/L of sodium bicarbonate (Sigma-Aldrich, St. Louis, MO) and 20 mM of HEPES (Sigma-Aldrich, St. Louis, MO) were added to PBS, and 2% w/w of Pluronic F-68 (Sigma-Aldrich, St. Louis, MO) was added to PBS to create PBS+Shear Protectant. To prepare diluted samples, all diluents were allowed to reach room temperature (18 to 22°C) before adding 1 part of cell culture to 4 parts of diluent (5 parts total, 1:5 dilution). Samples were diluted under a non-sterile condition. The diluted samples were either counted immediately, or incubated for a prescribed time to ascertain the effect of sample incubation time on viability. The sample incubation was also non-sterile, and the samples were open to the environment at room temperature.

### Microbioreactor vs. 3 L bench-top systems

For the MBR (ambr15^TM^, Sartorius AG, Gottingen, Germany) and 3 L bioreactor (Applikon Inc., Foster City, CA) comparison experiments, two cell lines (Cell Line A and B) were evaluated using a proprietary fed-batch process. Cells were cultured in a commercially-available proprietary basal medium. Seattle Genetics proprietary feed medium was added to the culture at various concentrations on selected culture days. Process parameters such as pH, dissolved oxygen, temperature and agitation were controlled.

For 3 L bioreactor system, cell culture samples were manually drawn and prepared for cell counting one reactor at a time. Samples were diluted using PBS only when the TCC was greater or equal to 1x10^7^ cells/mL. Given that the samples were immediately counted after dilution, the PBS incubation time was minimized.

For the MBR system, all cell culture samples were collected and diluted using PBS regardless of the TCC by the liquid handler. For Experiment 1 and 3 (Fig 1A), the diluted samples were held in the collection area for at least 15 minutes before cell counting; whereas, diluted samples from Experiment 2 were counted immediately. In addition, antifoam was added to the MBR culture starting on day 0 to minimize foaming.

### Effect of sample handling, antifoam addition and dilution on cell viability

To investigate the cause of decreased cell viability for cells grown in the MBR, an experiment was set up to evaluate sample handling, antifoam addition and sample dilution using the MBR system. A single source of cells was grown in shake flask. On the day of experiment, the cells were transferred from the shake flask into different MBR vessels, and evaluation was performed over 2 days. For sample handling evaluation, cells were sampled either manually or by the MBR liquid handler (robot). The samples were counted without dilution. For antifoam evaluation, antifoam was added to the culture at approximately 10 ppm, and cells were sampled by the robot. The samples were counted without dilution. For sample dilution evaluation, cells were sampled by the robot, and were counted immediately with and without PBS dilution.

### Evaluation of alternative diluents

A single source of cells, which were grown in a shake flask, was used for evaluating alternative dilutents. On the day of experiment, cells were manually sampled and diluted with diluent. For each counting session, 12 samples were prepared at the same time. Among 12 samples, 4 of the samples were neat samples (control) and the remaining 9 samples were test condition. Since the automated cell counter sample tray can only fit 9 samples at a time, 9 samples were loaded with the first 3 samples being the neat samples. As the sample queue reduced, more samples were loaded with the last (12^th^) sample being the neat sample.

### Evaluation of automated cell counter post mixing steps

All reagents such as Trypan blue, cleaning agent, buffer solution and disinfectant (P/N: 383722, Beckman Coulter, Brea, CA) used in the automated cell counter were replaced and primed with either PBS or PBS+Shear Protectant. A single source of cells was used for this experiment. Each sample was diluted and loaded on to the Vi-Cell and processed through the machine one at a time. Immediately after cell counting, the flow-through was collected from the waste bottle. The flow-through sample was then loaded onto a chemical analyzer to measure LDH.

### Evaluation of incubation time and total cell counts

Cells cultured in several shake flasks were allowed to grow to different VCD’s in order to obtain different TCC’s. On the day of cell counting session, samples were taken from different cell sources, and diluted using different diluents. The diluted samples were allowed to incubate for the prescribed times from 0 up to 55 minutes before being counted with an automated cell counter.

### Sample analysis

Automated cell counts and viability were obtained by the Trypan blue-dye exclusion microcopy using an automated cell counting system (Vi-Cell XR, Beckman Coulter, Brea, CA). The default setting of the machine was 50 images with 1x sample and 3x Trypan-blue dye mixing cycles unless otherwise specified. All cell counters were on manufacturer preventative maintenance schedule for every 6 months. The control beads (P/N: 175478, Beckman Coulter, Brea, CA) were run every two weeks, and the viability beads (0, 25, 50, 75 and 100%) (P/N: 24622, 25997, 24623, 24624, 24626, Polysciences, Inc., Warrington, PA) were run every 3 months to ensure optimal automated cell counter performance, counting accuracy and precision. In addition, cell counting images were randomly selected to evaluate accuracy of results.

Manual cell counts were performed by a well-trained, experienced analyst using a hemocytometer. Briefly, cell samples, whether neat or diluted, were gently mixed with trypan blue dye prior to loading the counting chamber. Cells in the large squares at the four corners were counted, and the cells that touched the lines were not counted. The total cell numbers were calculated based on the following equation:

$$\frac{Total\;cells}{mL}=\frac{Total\;cells\;counted\times dilution\;factor\times 10,000\;cells/mL}{Number\;of\;squares\;counted}$$

Lactate dehydrogenase (LDH) was measured by using a chemical analyzer (Cedex BioHT, Roche Diagnostics GmbH, Mannheim, Germany).

### Data analysis

To standardize the inter-experimental variability, the viability difference was used instead of viability. The viability difference was calculated by subtracting the viability of the test condition from the average viability of the control condition. The control condition is the neat sample unless otherwise specified. A viability difference of 0 indicates both the test and average control conditions had the same viability. A number that is farther away from 0 in the positive or negative direction indicates the test condition has either higher or lower viability, respectively, than the average control.

Statistical analysis was performed by using the statistical software (JMP) (SAS Institute Inc., Cary, NC) to compare the means and standard deviations of different conditions as well as to generate a statistical model.

## Results

### PBS dilution decreases viability

A discrepancy of viability was first observed when comparing experimental results obtained with an MBR system and those obtained with 3 L bioreactor systems. The viability results from three independent experiments are shown in Fig 1A. The average viability difference for both experiments 1 and 3 was 4.5±4.1% with the maximum viability difference of 14.9%; whereas, the average viability difference for experiment 2 was 1.1±1.5%. Cell culture samples from the 3 L bioreactors were only diluted with PBS when the VCD exceeded 1x10^7^ cells/mL, and all samples from MBR were diluted with PBS. Furthermore, samples from the 3 L bioreactors were counted immediately after sampling (and dilution if required) to minimize the incubation time; whereas, samples from the MBR in experiments 1 and 3 were held for at least 15 minutes after dilution before loading onto the cell counter, and samples from experiment 2 were counted immediately after dilution. To quantify the variability, the daily standard deviations (STDEV) of viability from each experiment were compared between systems (Fig 1B). The means of the daily STDEV of viability from experiments 1 and 3 were statistically higher (p<0.05) in the MBR than the 3 L bioreactor.

**Fig 2:**
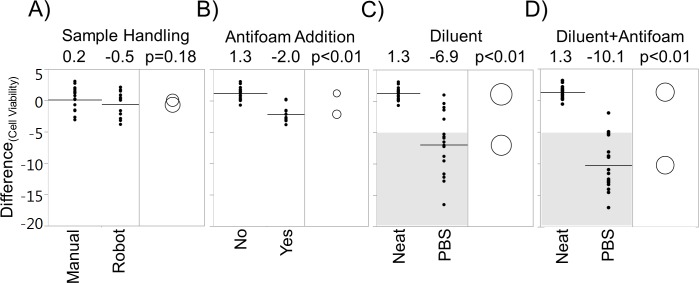
Investigation of lower viability. (A) Sample handling (manual, n = 17 and robot, n = 12). (B) Antifoam addition (no, n = 19 and yes, n = 18). (C) Diluent (Neat, n = 17 and PBS, n = 19). D) Additive effect of diluent and antifoam (neat, n = 12 and PBS, n = 18). Raw data included in S1 Dataset.

Variation in sample handling, differences in the antifoam addition during cell culture, and whether or not a diluent was used were identified to be potential causes of the apparent low viability observed in the MBR system. The Tukey’s test revealed that the p-values for sample handling, antifoam addition and PBS dilution were 0.18, < 0.01 and < 0.01, respectively (Fig 2A, 2B and 2C). The difference of the means in viability between with (n = 19) and without (n = 18) antifoam conditions was 3.3% (Fig 2B); whereas, the difference was 8.2% between neat (n = 17) and PBS diluted (n = 19) samples (Fig 2C). Furthermore, the effects were additive, and the difference in viability of PBS diluted sample with antifoam was 11.4% when compared to the neat sample without antifoam addition (Fig 2D).

**Fig 3:**
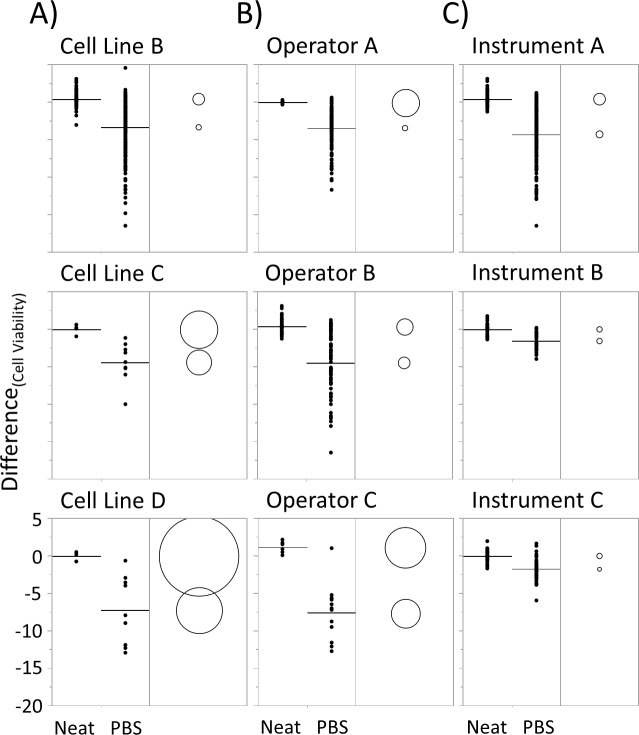
Effect of PBS is independent of A) cell line, B) operator and C) cell counter. p < 0.05 for all conditions. Raw data included in S1 Dataset.

While the mean effect of PBS dilution was -6.9% on viability, the majority (>80%) of data points ranged between -5% and -17%; whereas, none of the control conditions had viability difference below -5% (Fig 2C). In addition, the effect of PBS on viability is independent of cell line, operator and cell counter station as PBS-diluted samples consistently have reduced viability across multiple cell lines, operators and automated counters (Fig 3).

### Fresh medium or pbs with shear protectant preserves the viability

**Fig 4:**
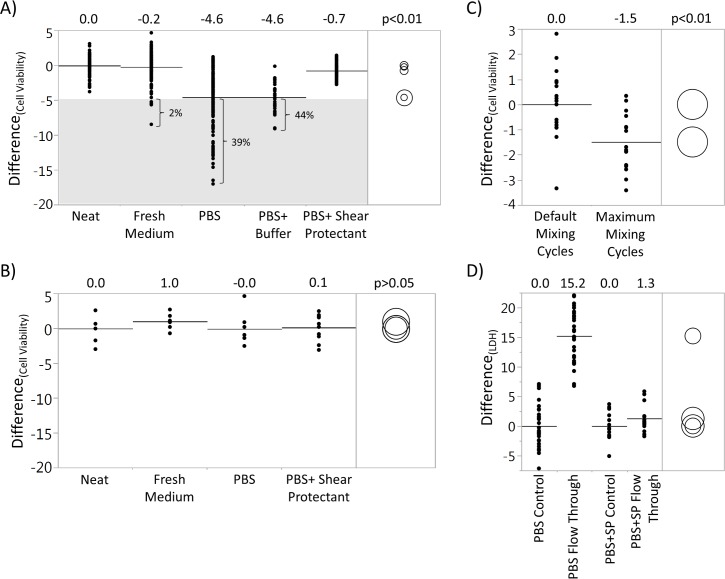
Alternative diluents. A) Data collected from automated cell counters. Neat (n = 114), fresh medium (n = 258) and PBS+Shear Protectant (n = 126) are not statistically different comparatively, but, are statistically different than both PBS (n = 229) and PBS+Buffer (n = 32). B) Data collected from manual cell counts (hemocytometer). All diluents are not statistically different (n = 7 for neat, fresh medium and PBS, and n = 10 for PBS+Shear Protectant). C) Viability difference between mixing cycles using PBS as the diluent (n = 18). D) Difference in LDH before (control) and after cell counting procedures (flow through) between PBS (n = 28) and PBS+Shear Protectant (n = 14). Raw data included in S1 Dataset.

An experiment was conducted using medium (n = 258), PBS (n = 229), PBS containing biological buffers (PBS+Buffer, n = 32) and PBS containing poloxamers (PBS+Shear Protectant, n = 126) as diluents for cell culture samples, and neat samples (n = 114) were the control (Fig 4). For samples analyzed on an automated cell counter, the Tukey’s test revealed that the neat samples and samples diluted using medium and PBS+Shear Protectant were not statistically different comparatively but were statistically different (p<0.01) than the samples diluted using PBS and PBS+Buffer (Fig 4A). The average viability differences were 0.0% (neat), -0.2% (medium), -4.6% (PBS), -4.6% (PBS+Buffer), and -0.7% (PBS+Shear Protectant). Although the mean difference in viability of the PBS dilution condition was not as profound as observed in the previous experiment (-4.6% vs. -6.9%), the large spread of data points (18.2% vs. 18.0%) remained the same. About 40% of the data points were below -5% for both PBS and PBS+Buffer conditions.

### Shear stress is likely introduced during automated cell counting procedures

Neat samples and samples diluted in fresh medium, PBS and PBS+Shear Protectant were manually counted using a hemocytometer (Fig 4B). Each condition had at least 7 replicates, and the actual cell counts were between 150 and 300 to ensure accuracy and precision. The Tukey’s test demonstrated that none of the conditions were statistically different (p>0.05). The differences in viability were 0.0, 1.0, 0.0 and 0.1% for neat, fresh medium, PBS, and PBS+Shear Protectant, respectively. This indicates that the variability is associated with some aspect of the automated counting procedure rather than anything intrinsic to the sample preparation.

Experiments were conducted to examine whether shear stress on cells was associated with the observed variability, and where the shear stress was encountered during the automated cell counting procedures. The first experiment was focused on the mixing cycles of the samples. Both sample and Trypan blue dye mixings were set to the maximum of 9 cycles. PBS-diluted samples (n = 18) were counted and the results were compared to the default mixing cycles. TCC, viable cell counts and viability differences between the two conditions were all statistically significant (p<0.05). The viability difference of the maximum mixing cycle condition was about 1.5% lower than the default condition (Fig 4C).

The next experiment examined the post-mixing steps for potential introduction of shear stress. The automated cell counter reagents were switched and primed with the diluent of interest. After cell counting procedures, the flow through sample was collected in a clean waste bottle, and LDH was analyzed as it is indicative of cellular damage especially from shear stress and is an orthogonal method to trypan blue dye-exclusion method. The control represents samples that did not go through the cell counting procedure. The “flow through” represents samples that did go through the cell counting procedure. The average LDH differences between the controls and flow through were about 15.2 u/L (p<0.05) and 1.3 u/L (p>0.05), with a relative differences of 11% and 1%, for samples diluted in PBS and PBS+Shear Protectant, respectively (Fig 4D).

### Effect of incubation time and total cell counts

**Fig 5:**
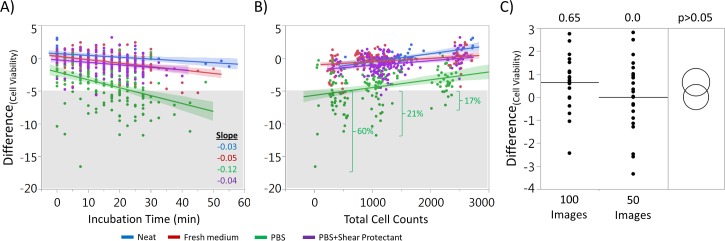
Bivariate plots of viability differences vs. (A) incubation time and (B) TCC. (C) Increasing TCC by adjusting image captures (50 images, n = 25; 100 images, n = 21). Raw data included in S1 Dataset.

To elucidate the cause of the large spread of reported viability seen with the PBS diluted samples, the data were analyzed, and both diluent incubation time and TCC were identified as statistically significant (p<0.01) factors. An experiment was then conducted to further investigate these two factors. Four conditions were tested: neat, fresh medium, PBS and PBS+Shear Protectant. Daily cell culture samples were incubated in different diluents, or neat, for up to 55 minutes before counting on the automated cell counter. Trending data were created by fitting a linear regression to data in a bivariate fit graph comparing incubation to viability difference for each condition (Fig 5A). For the effect of diluent incubation time, the slopes were -0.03 (neat), -0.05 (fresh medium), -0.12 (PBS) and -0.04 (PBS+Shear Protectant) (Fig 5A). Although a slope of -0.12 does not seem to be a significant drop over time (about 4% viability over 30 minutes of incubation time), 33% of the PBS data were below -5% viability difference; whereas, only 3 out of 311 data points from the rest of conditions combined were below -5%.

The data from the incubation time experiment were used to generate bivariate fit plots between TCC and viability differences (Fig 5B). Linear regression was fitted to all data to provide trending information. It was observd that the viability differences decreased when TCC was increased. The percentages of data points that have a viability difference greater than -5% for PBS condition were 60% when the TCC is less than 600, 21% when the TCC is between 600 and 2000, and 17% when the TCC is greater than 2000. A TCC of 2000 roughly translates to 3x10^6^ cells/mL for a neat sample and 15x10^6^ cells/mL when the sample is 1:5 (5 parts total) diluted. Collectively, about 33% of the data points were below -5% for the PBS condition. The correlation between viability drops and TCC is not due to the number of cells analyzed as there is no statistically significant difference between 50 and 100 images (Fig 5C).

## Discussions

A critical issue caused by cell culture sample dilution using PBS in conjunction with the commercially available cell counter was identified. This phenomenon was first observed in a 3 L bioreactor and MBR comparison study (Fig 1). Significant discrepancy of viability between the two systems was observed in experiments 1 and 3. Furthermore, the daily viabilities among replicates (n = 3) within the same experiment (1 and 3) were more variable in the MBR system. Since the drop in viability was observed shortly after dilution and cell counting, the cell death was hypothesized to be necrotic. Factors such as sample handling, antifoam addition and sample dilution were examined, and both antifoam addition and PBS dilution were identified to lower the viability (Fig 2). Daily antifoam addition to the MBR is necessitated by the relatively small headspace of the MBR. High concentration of antifoam has been shown to be detrimental to cell growth [15]. Although antifoam addition had negative impact to the viability, the effect was small compared to that of the PBS dilution (-3.3% vs. -8.2%). Furthermore, the large spread of data for the PBS dilution condition corroborated with the MBR experiment. This validates that cell culture samples diluted in PBS have lower and more variable viability measurements than the control (Fig 2C). Cell culture sample dilution is sometimes necessary to preserve the working volume and/or to extend assay measurement range. Therefore, it is imperative to identify alternative diluents that do not inadvertently decrease viability.

It was hypothesized that since the neat samples did not exhibit a decrease in viability, some aspect of the diluent might be responsible. A variety of alternative diluents were explored. PBS lacks nutrients, so growth media was tested for its ability to preserve viability. Furthermore, besides lacking essential nutrients, PBS does not contain significant buffer capacity, so PBS with additional buffer capacity was tested. A third diluent, PBS containing anti-shear additives, was also tested, based on the possibility that shear stress on cells was responsible for the low viability. The PBS+Buffer condition did not preserve the viability suggesting that the cell death was not due to pH fluctuations (Fig 4A). In contrast, both medium and PBS+Shear Protectant can be used as alternative diluents which would not inadvertently decrease the viability.

Given that PBS+Shear Protectant was able to preserve the viability, it is believed that shear stress was introduced during the process of diluting and counting cells. Poloxamers have long been used in cell culture medium as a shear protectant. The profound effects of shear stress on cells such as losing adhesion, productivity and viability have been well documented [16–19]. In sparged cultivation systems, the protective effect of poloxamers is believed to modify the liquid-gas interface with surface-active components which prevent direct contact of the cells to the bubbles [20]. In addition, poloxamers also directly modify the cell membrane by decreasing its porosity or plasma membrane fluidity, and is able to maintain cell membrane integrity under high shear stress conditions [21]. Further investigations revealed that the automated cell counter is likely introducing shear stress during the counting process which inadvertently damages the cells, and the poloxamers in both fresh medium and PBS+Shear Protectant were able to protect the cells from shearing (Fig 4B, 4C and 4D).

The adverse effect of PBS diluted sample was incubation time and TCC dependent, and the viability could be lowered by as much as 17% when compared to non-diluted samples.

It is hypothesized that the poloxamers, which typically are present in cell culture media, desorb from cell membranes over time when the sample is diluted by PBS. Therefore, the cells become more sensitive to shear stress as the incubation time increases. Besides poloxamers, sera and naturally secreted proteins have been shown to have similar effect as shear protectants [21,22]. It is conceivable that the effect of TCC on viability is due to the presence of these natural shear protectants. At the low TCC and high viability condition, the concentration of the secreted proteins is lower when compared to high TCC and either high or low viability conditions.

Although the impact of PBS dilution might not be critical in the manufacturing environment since samples are typically few and, in some cases, counted immediately, the issue can be more serious in the process development setting especially when the MBR system is used and the process has relatively low peak VCD. As MBR systems become increasingly popular in the process development setting by enabling exploration of multiple conditions in a single experiment, daily sample analyses can be a rate-limiting step due to lack of high-throughput alternatives for certain assays. Samples are often diluted using PBS to compensate for the small working volume of MBR, and are often queued up for cell counts. The time between the first and last sample counted could potentially be greater than an hour. In this case, the cell viability could be significantly impacted if diluted using PBS. Furthermore, the impact of PBS dilution (1:5) is more significant when the peak VCD is lower than 15x10^6^ cells/mL for a given process. In this case, the majority of the TCC will likely fall under 2,000 counts, and data previously mentioned suggests a significant portion of the measurements will have lower viability than what the actual sample should be.

## Conclusions

Viability measurements are fundamental to cell culture process development and manufacturing of therapeutic proteins. A systematic investigation has revealed that cell culture samples diluted in PBS could inadvertently lower the viability when measured using automated cell counters. The effect of PBS on viability can be consistently reproduced, and is independent of process scale, cell line, operator and automated cell counter used. In addition, the negative impact of PBS on the viability is proportional to the sample incubation time, and inversely proportional to the TCC. The cause of lower viability was attributed to shear stress introduced during the sample preparation procedure by the automated cell counter. Alternative diluents were identified to remedy this issue.

## Supporting information

S1 Dataset Raw data. (XLSX)
